# *Enterococcus faecium* multicenter antimicrobial resistance surveillance in Tunisia (2010-2022): high vancomycin resistance rates and linezolid resistance emergence

**DOI:** 10.4314/ahs.v26i2.5

**Published:** 2026-06

**Authors:** Anis Raddaoui, Mouna Louisa Ben Moussa, Yosra Chebbi, Siwar Frigui, Sadok Bougharriou, Lamia Kanzari, Khaoula Meftah, Hajer Battikh, Ons Haddad, Sarra Dhraief, Salma Kaoual, Yassmine Maatouk, Khouloud Ben Dhaou, Manel Hamdoun, Senda Mezghani, Faouzia Mahjoubi, Yosr Kadri, M Marzouk, A Ghariani, Farouk Barguellil, Olfa Bahri, Jalel Boukadida, Neila Hannachi, Sophia Besbes, Lamia Thabet, Maha Mastouri, Meriem Zribi, Hanen Smaoui, Leila Slim, Emna Mhiri, Ilhem Boutiba-Ben Boubaker, Adnene Hammami, Hela Karray, Ramzi Ouhichi, Wafa Achour

**Affiliations:** 1 Service des Laboratoires, Centre National de Greffe de Moelle Osseuse- LR18ES39, Faculté de Tunis, Tunisie; 2 Service de Microbiologie, Hôpital Habib Bourguiba, Sfax, Tunisie Université de Sfax, Tunisie; 3 Hôpital Charles Nicole, Laboratoire de Microbiologie, Tunis, Tunisie - Université Tunis El Manar, Laboratoire de recherche, LR99ES09, Faculté de Médecine de Tunis, Tunisie; 4 Service de Microbiologie, Hôpital d'Enfant Bechir Hamza, Tunis, Tunisie; 5 Service de Microbiologie, Hôpital La Rabta, Tunis, Tunisie; 6 Service de Microbiologie, Hôpital Fatouma Bourguiba, Monastir Laboratoire de recherche des maladies transmissibles et substances biologiquement actives LR99ES27, Faculté de pharmacie Université de Monastir, Tunisie; 7 Service des Laboratoires, Centre de Traumatologie et des Grands Brulés, Ben Arous, Tunisie; 8 Laboratoire de Biologie Clinique, Institut Mohamed Kassab d'Orthopédie, Manouba, Tunisie; 9 Service de Microbiologie, Hôpital Farhat Hached, Sousse, Tunisie; 10 Service de Microbiologie, Hôpital de Pneumophtysiologie Abderrahmen Mami, Ariana, Tunisie; 11 Service de Microbiologie-Biochimie, LR16SP01, Hôpital Aziza Othmana, Tunis, Tunisie; 12 Service de Microbiologie, Hôpital Militaire Principal d'Instruction de Tunis, Tunisie; 13 Tunisia World Health Organization Country Office

**Keywords:** *Enterococcus faecium*, vancomycin resistance, linezolid resistance

## Abstract

**Background:**

Vancomycin-resistant *Enterococcus faecium* is classified among World Health Organization priority pathogens for Research and development of new antibiotics. This study presents the results of 13-year surveillance of antibiotic resistance in *E. faecium*, with a specific focus on vancomycin and linezolid resistance in thirteen university hospitals across various regions of Tunisia.

**Methods:**

All participating laboratories followed a standardized methodology for clinical sample culture, bacterial identification, antibiotic susceptibility testing according to CA-SFM guidelines, result interpretation, epidemiological data collection. Additionally, they implemented quality controls.

**Findings:**

During the surveillance period, 2,931 *E. faecium* isolates were collected, predominantly from urine (49.1%) and blood cultures (24%), with high prevalence in Surgery Departments (30.3%) and Intensive Care Units (23.6%). Resistance rates were high for ampicillin (87.1%), erythromycin (93.6%), gentamicin (62.8%) and vancomycin (26%). Since 2019, six linezolid-resistant isolates have been isolated and only one isolate resistant to tigecycline was identified in 2021. Vancomycin resistance had significant upward trend since 2012, peaking at 38.1% in 2017. It was significantly associated to gentamicin (84.9% vs. 53.8%) (p<0.0001) and erythromycin95.8% vs. 89.6%) (p=0.0001) in a five year period (2018 - 2022).

**Conclusion:**

This worrying situation requires deeper investigations to develop adapted strategies with targeted measures in high-risk departments.

## Introduction

Enterococci are commensal bacteria that colonize the human gastrointestinal tract and possess a remarkable ability to survive in hostile environments, including hospital settings^1^,^2^. These organisms are significant pathogens in healthcare, particularly implicated in infections such as endocarditis and urinary^2^. This multicenter study. Notably, Enterococcus faecalis (*E. faecalis*) and *Enterococcus faecium* (*E. faecium*) are responsible for over 90% of enterococcal infections^3^. *E. faecalis* is considered the most virulent species, accounting for approximately 85% of these infections, while *E. faecium* is recognized as the most antibiotic-resistant species, posing a significant challenge in clinical settings^1^,^2^.

Enterococci exhibit low-level resistance to aminoglycosides, as well as resistance to sulfonamides and cephalosporins. Additionally, *E. faecium* frequently exhibits resistance to ampicillin^4^. The treatment of infections caused by *E. faecium* generally relies on the use of glycopeptides^2^,^4^. Glycopeptide resistance in *E. faecium* emerged in 1986 and subsequently spread worldwide. Over the past two decades, the prevalence of this resistance has rapidly increased in several countries, rising from less than 10% to over 40%^5^,^6^. This resistance is often observed in patients with a history of multiple antibiotic use and prolonged hospitalization^5^ and has been associated with increased morbidity and mortality from these infections^1^,^2^. In 2017, the World Health Organization listed vancomycin-resistant *Enterococcus faecium* (VREfm) as a high-priority pathogen, following carbapenem-resistant Acinetobacter baumannii and Pseudomonas aeruginosa, and carbapenem- and third-generation cephalosporin-resistant Enterobacteriaceae^7^.

In Tunisia, VREfm was first isolated in 2003, with a significant spread observed from 2012 onwards^8^. Since then, limited data on E. faecium and VREfm has been reported in Tunisia. This study presents the results of 13-year surveillance (2010–2022) of antibiotic resistance in *E. faecium*, focusing particularly on vancomycin resistance in thirteen Tunisian university hospitals across different regions.

## Methods

Our study included all *E. faecium* isolates responsible for infections isolated between 2010 and 2022 from the laboratories of thirteen Tunisian university hospitals with a total of 5656 beds: Charles Nicolle Hospital (Tunis), Bechir Hamza Children's Hospital (Tunis), La Rabta Hospital (Tunis), National Bone Marrow Transplant Center (Tunis), Aziza Othmana Hospital (Tunis), Principal Military Hospital of Instruction (Tunis), Abderrahmen Mami Pneumo-Phtysiology Hospital (Ariana), Mohamed Kassab Institute of Orthopedics (Manouba), Traumatology and Major Burns Center (Ben Arous), Fatouma Bourguiba Hospital (Monastir), Farhat Hached Hospital (Sousse), Habib Bourguiba Hospital (Sfax) and and Hedi Chaker Hospital (Sfax).

This study is based on the results of antibiotic resistance surveillance conducted by the research laboratory ‘Laboratory of Antibiotic Resistance’. All laboratories participating in the surveillance program adopted a similar methodology regarding clinical samples culture, bacterial identification (phenotypic and biochemical methods), antibiotic susceptibility testing according to the guidelines of the Antibiogram Committee of the French Society of Microbiology (CA-SFM) guidelines^9^, quality controls, result interpretation, epidemiological data collection, duplicate elimination, and data stratification.

### Statistical Analysis

Comparison of percentages on independent series was performed using Pearson's chi-square test. Temporal trend analysis was conducted using Spearman's rank correlation test (RS). The significance threshold (p) was set at 0.05 for all statistical tests.

### Ethics approval

This study was conducted with the approval of the Local Medical Committee for National Bone Marrow Transplantation, under reference number CNGMOEC-0224.

## Results

A total of 2,931 *E. faecium* isolates were isolated during the study period. The number of isolates significantly increased from 82 in 2010 to 287 in 2022 (Rs = 0.79, p = 0.002). The isolates were primarily isolated from urine samples (49.1%) and blood cultures (24%). They were particularly prevalent in Surgery Departments (30.3%), Intensive Care Units (23.6%), and Pediatrics Departments (17.6%) (Table 1).

**Table 1:** Distribution of *E. faecium* Strains by Department and Specimen Collection

	Rate
**Department**	
**Surgery**	30.3%
**Intensive Care**	23.6%
**Pediatrics**	17.6%
**Medicine**	13.5%
**Oncology-Hematology**	4.6%
**Ambulatory**	5.6%
**Neonatology**	3.8%
**Gynecology**	1%
**Specimen**	
**Urine**	49.1%
**Blood Cultures**	24%
**Pus**	16.6%
**Biopsies**	3.9%
**Respiratory**	1.2%
**Other**	4.9%

The study of antibiotic resistance revealed that *E. faecium* isolates exhibited high resistance rates, with ampicillin resistance reaching 87.1%. Notably, there was a significant upward trend in ampicillin resistance, increasing from 75.6% in 2010 to 95.7% in 2022 (Rs = 0.769, p = 0.002). Similarly, a rapid and significant increase in resistance to pristinamycin was observed (Rs = 0.863, p < 0.001), rising from 8.5% in 2010 to 57.8% in 2022. In contrast, erythromycin showed an overall resistance rate of 93.6%, with a non-significant trend towards a decrease, from 96.3% in 2010 to 88% in 2022 (Rs = -0.517, p = 0.07) (Table 2). During this study period, only six isolates resistant to linezolid were identified in 2019 and 2022, and only one isolate resistant to tigecycline was identified in 2021.

**Table 2:** Yearly Antibiotic Resistance Rates in *E. faecium*

	Total	2010	2011	2012	2013	2014	2015	2016	2017	2018	2019	2020	2021	2022	Rs	p
**Amoxicillin**	87.1%	75.6%	84.1%	85.2%	81.7%	78.4%	82.4%	90.3%	91.1%	91.1%	84.1%	89.6%	94.8%	95.7%	0.769	0.002
**Gentamicin**	62.8%	50%	70%	61.5%	57.1%	44.1%	54.9%	69.4%	56.6%	60.4%	66.7%	62.4%	70.1%	68.8%	0.440	0.135
**Erythromycin**	90.8%	96.3%	95.9%	91.7%	90.9%	87.7%	89.3%	97%	94.4%	93.1%	90.4%	77%	90.4%	87.9%	-0.517	0.070
**Pristinamycin**	35.2%	8.5%	0%	7.1%	9.1%	7.8%	8.4%	35.1%	48.5%	47.5%	60.1%	48.3%	53.8%	57.8%	0.863	0.0001
**Vancomycin**	26%	0%	0%	17.2%	27.2%	21.8%	26.2%	37.2%	38.1%	22.6%	26.2%	30.1%	29.9%	29.4%	0.680	0.01

High-level resistance to gentamicin was observed at an overall rate of 62.8%, demonstrating notable stability over the years, with the exception of two peaks recorded in 2011 (70%) and 2021 (70.1%) (Table 2).

With regard to vancomycin, 26% of the isolates were found to be resistant, showing a significant upward trend (Rs=0.680, p=0.01) since its emergence in 2012, marked by two peaks in 2016 (37.2%) and 2017 (38.1%). The study of associated resistance during the last five years (2018-2022) revealed significantly higher resistance rates among vancomycin resistance isolates, compared to vancomycin susceptible ones, to gentamicin (84.9% vs. 53.8%) (p<0.0001) and erythromycin (95.8% vs. 89.6%) (p=0.0001) (Table 3).

**Table 3:** Antibiotic Resistance Rates in Vancomycin-Resistant *E. faecium* (2018–2022)

	*E. faecium*		
	Vancomycin-Resistant (n= 400)	Vancomycin-Sensitive (n= 1056)	p
**Gentamicin**	84.9%	53.8%	<0.0001
**Erythromycin**	95.8%	89.6%	0.0001
**Pristinamycin**	41.4%	41.2%	0.915
**Linezolid**	0.8%	0.2%	0.355

## Discussion

This multicenter study is the largest and longest of its kind conducted in Tunisia, encompassing thirteen major university hospitals over a 12-year period, and focusing on 2,931 *E. faecium* infectious isolates. Studies on the epidemiology of *E. faecium* and VREfm in Tunisia are scarce, with only four studies available, each covering a single university hospital^10^,^11^,^12^,^13^.

The majority of *E. faecium* isolates originated from urine and blood cultures, particularly from the Surgery, Pediatrics, and Intensive Care Units. Digestive surgery and invasive urinary catheterization procedures facilitate infection by this bacterium, which colonizes the digestive tract and can translocate to the blood through contiguity and digestive translocation^2^,^14^. Additionally, antibiotic use in these departments disrupts the microbiota, favoring the colonization by antibiotic-resistant *E. faecium*^2^,^5^.

The ampicillin resistance rate was high (87.1%) and stable among *E. faecium* isolates. Similar rates are reported, in 2019, in Belgium (86%), Italy (89.3%), and Spain (86.9%)^15^. Indeed, *E. faecium* exhibits natural resistance to cephalosporins due to the presence of a low-affinity Penicillin-Binding Protein 5. Additionally, mutations in this PBP (PBP5′), with or without hyperproduction, lead to ampicillin resistance^3^,^4^.

*E. faecium* isolates showed a very high overall resistance rate to erythromycin (90.8%). A meta-analysis compiling results from 26 international studies showed an erythromycin resistance rate of 84%^16^. Molecular studies of macrolide resistance in enterococci isolated in Tunisia reveal that this resistance is mainly encoded by the ermB gene^11^,^12^,^13^. This gene is mobilized by several transposons (Tn1545/916, Tn917, and Tn3871), facilitating its transfer among Gram-positive cocci^17^. A meta-analysis of 34,487 Gram-positive cocci strains isolated in Africa showed the predominance of ermB and ermC genes among macrolide-resistant strains^18^.

The overall rate of high-level gentamicin resistance remained both high and stable (63.6%). Similarly, Belgium has reported persistently elevated rates, increasing from 52.9% in 2008 to 67.7% in 2019. In contrast, Latvia has experienced a marked rise, with resistance rates escalating from 25% in 2013 to 61.8% in 2019 and reaching 100% by 2022^15^. In Tunisia, a national survey conducted in 2012 reported that aminoglycosides were the fifth most frequently used class of antibiotics for treating nosocomial infections, following β-lactams, quinolones, and glycopeptides, accounting for 9.7% of all antibiotics used. Resistance to aminoglycosides is primarily mediated by the aac(6′)-aph(2″) gene, which is commonly carried on the transposon Tn4001, as demonstrated in previous Tunisian studies^11^,^12^,^13^.

The overall vancomycin resistance rate was 26%. This rate places Tunisia among the countries with high vancomycin resistance prevalence, unlike several European countries that display rates below 5%, such as Belgium, France, and Spain^15^. However, other countries as Cyprus, and Latvia exhibit very high rates, reaching up to 60%^15^. These variations in resistance rates from one country to another may be due to differences in antibiotic consumption and to clonal spread of competitive and persistent clones^1^,^2^. In Tunisia, glycopeptide consumption accounts for 10.4%, ranking third after β-lactams and quinolones in the treatment of nosocomial infections^19^. Molecular typing of certain VREfm strains isolated in Tunisia revealed a clear predominance of the Clonal Complex 17 (CC17) (including ST18, ST80, and ST117). This clone is an internationally recognized pandemic strain resistant to vancomycin^11^,^13^, which may account for the peaks observed in 2016 and 2017. Regarding the genetic basis of this resistance, Tunisian studies have consistently reported the presence of the vanA gene carried on the Tn1546 transposon, which has been previously detected in the country^11^-^13^.

VREfm isolates exhibited significantly higher resistance rates to gentamicin and erythromycin than vancomycin-sensitive isolates. Similar results are noted in several other studies^13^. Indeed, VREfm can acquire mobile genetic elements, facilitating its adaptation to the hospital environment. Moreover, VREfm strains belonging to the CC17 clone exhibit high antibiotic resistance rates^20^.

VREfm was mainly isolated from blood cultures (35%) and urine samples (14.4%). VREfm strains belonging to the CC17 frequently carry the esp gene, which promotes biofilm formation on invasive devices such as catheters and tubes, thus favoring colonization and infection^6^. Additionally, VREfm bacteremia occurs in 34% of patients after 35 days of colonization by VREfm^6^.

Linezolid resistance is exceptionally rare among *E. faecium* strains and was first reported in Tunisia in 2019, more than a decade after the introduction of this antibiotic. Globally, linezolid resistance remains uncommon, generally not exceeding 2% in most countries^21^. This resistance is mainly mediated by mutations or the acquisition of transferable resistance genes^22^. In Africa, acquired linezolid resistance has been reported only in a few studies. In Tunisia, the optrA gene was initially detected in *E. faecalis* isolates from wastewater and later in *E. faecalis* and *E. faecium* strains of animal and food origin, some of which also harbored the poxtA gene^23^. In clinical settings, linezolid resistance has been associated with the presence of the optrA gene in *E. faecalis*^24^. These genes are mobilized by various mobile genetic elements such as plasmids and transposons, necessitating active surveillance.

Considered a last-line treatment for multidrug-resistant infections, tigecycline is frequently used to treat complicated skin and soft tissue infections and intra-abdominal infections. Since 2007, a progressive increase in tigecycline-resistant Enterococcus has been reported, but it remains low worldwide, with rates ranging from 0.3% in the American continent to 3.9% in the European continent^21^. Acquired tigecycline resistance in *Enterococcus faecium* is mainly mediated by mechanisms that reduce the intracellular concentration of the antibiotic or limit its binding to the ribosome. This resistance most frequently results from the overexpression of efflux pumps such as Tet(L), the production of the ribosomal protection protein Tet(M), or ribosomal genes that are selected under tigecycline exposure^25^. Consequently, a rigorous strategy to control tigecycline consumption should be implemented to mitigate resistance development.

## Conclusion

This study demonstrated the increasing isolation of VREfm, with emergence of linezolid and tigecycline resistance among these isolates. A study examining the correlation between vancomycin consumption and resistance rates, along with an exploration of the molecular epidemiology of VREfm isolates, is essential for identifying the diffusion of clonal strains or mobile genetic elements. This will help implement specific control measures in high-risk departments.
